# Virtual Integration Environment as an Advanced Prosthetic Limb Training Platform

**DOI:** 10.3389/fneur.2018.00785

**Published:** 2018-10-17

**Authors:** Briana N. Perry, Robert S. Armiger, Kristin E. Yu, Ali A. Alattar, Courtney W. Moran, Mikias Wolde, Kayla McFarland, Paul F. Pasquina, Jack W. Tsao

**Affiliations:** ^1^Walter Reed National Military Medical Center, Bethesda, MD, United States; ^2^Applied Physics Laboratory, Johns Hopkins University, Laurel, MD, United States; ^3^Henry M. Jackson Foundation, Bethesda, MD, United States; ^4^School of Medicine, University of California, San Diego, La Jolla, CA, United States; ^5^Uniformed Services University of the Health Sciences, Bethesda, MD, United States; ^6^University of Tennessee Health Science Center, Memphis, TN, United States

**Keywords:** upper extremity amputation, myoelectric prostheses, virtual integration environment, surface electromyography (semg), pattern recognition control, virtual reality therapy, modular prosthetic limb, neurorehabilitation

## Abstract

**Background:** Despite advances in prosthetic development and neurorehabilitation, individuals with upper extremity (UE) loss continue to face functional and psychosocial challenges following amputation. Recent advanced myoelectric prostheses offer intuitive control over multiple, simultaneous degrees of motion and promise sensory feedback integration, but require complex training to effectively manipulate. We explored whether a virtual reality simulator could be used to teach dexterous prosthetic control paradigms to individuals with UE loss.

**Methods:** Thirteen active-duty military personnel with UE loss (14 limbs) completed twenty, 30-min passive motor training sessions over 1–2 months. Participants were asked to follow the motions of a virtual avatar using residual and phantom limbs, and electrical activity from the residual limb was recorded using surface electromyography. Eight participants (nine limbs), also completed twenty, 30-min active motor training sessions. Participants controlled a virtual avatar through three motion sets of increasing complexity (Basic, Advanced, and Digit) and were scored on how accurately they performed requested motions. Score trajectory was assessed as a function of time using longitudinal mixed effects linear regression.

**Results:** Mean classification accuracy for passive motor training was 43.8 ± 10.7% (14 limbs, 277 passive sessions). In active motor sessions, >95% classification accuracy (which we used as the threshold for prosthetic acceptance) was achieved by all participants for Basic sets and by 50% of participants in Advanced and Digit sets. Significant improvement in active motor scores over time was observed in Basic and Advanced sets (per additional session: β-coefficient 0.125, *p* = 0.022; β-coefficient 0.45, *p* = 0.001, respectively), and trended toward significance for Digit sets (β-coefficient 0.594, *p* = 0.077).

**Conclusions:** These results offer robust evidence that a virtual reality training platform can be used to quickly and efficiently train individuals with UE loss to operate advanced prosthetic control paradigms. Participants can be trained to generate muscle contraction patterns in residual limbs that are interpreted with high accuracy by computer software as distinct active motion commands. These results support the potential viability of advanced myoelectric prostheses relying on pattern recognition feedback or similar controls systems.

## Introduction

Following upper extremity (UE) loss, individuals face a variety of functional challenges that restrict their ability to complete activities of daily living and lead to decreased quality of life. By improving functionality, upper limb prostheses have the potential to improve quality of life for individuals with limb loss (1). Despite the promise that prostheses hold, the current rate of prosthetic usage among individuals with UE loss ranges from 27 to 56% (2, 3). Low rates of prosthetic use are largely explained by user dissatisfaction with existing protheses. An ideal prosthesis needs to replicate human hand features, incorporate into the user's sense of self, and operate by near-natural control (4). Currently, some of the most advanced prosthetic hands offer multiple, simultaneous degrees of freedom of motion via non-invasive methods like surface electromyography (sEMG). Control of such prostheses requires an operating system capable of discriminating between various sEMG signals and a user capable of sustaining the significant cognitive burden associated with pattern recognition control of a dexterous, myoelectric prosthesis (4). Therefore, there exists the need for the development of training platforms that pair with advanced prosthetic systems and lead to effective and intuitive prosthetic control.

There are few such platforms currently available for training with advanced prosthetic hands. Pons et al. (5) explored the use of virtual reality-based muscle conditioning and command language learning to teach users to control the MANUS, an UE prosthesis. In 2011, the Johns Hopkins University Applied Physics Laboratory (JHU/APL) created the Virtual Integration Environment (VIE)—a virtual reality simulator for UE prosthetic training (6). The VIE allows individuals with UE loss to both follow and command the movements of a virtual avatar using sEMG signals captured from their residual limbs (7, 8). Sophisticated software algorithms associate user-generated sEMG signal patterns with UE motions, allowing the participant to intuitively drive the movements of a virtual limb (9–11). The VIE was created in part as a screening and training device for an advanced myoelectric prosthetic developed by JHU/APL for DARPA Revolutionizing Prosthetics 2009 (12–14).

In this study, we evaluated the application of the VIE as a motor training tool with active duty military personnel with UE loss. Training and motion accuracy scores were collected and used to assess how pattern recognition and machine learning could lead to improved motor control within the virtual system.

## Materials and methods

### Participants

The data was collected as part of the clinical trial “Virtual Integration Environment in Decreasing Phantom Limb Pain,” identifier number NCT01462461 (ClinicalTrials.gov). Volunteers were recruited at Walter Reed National Military Medical Center (WRNMMC) in Bethesda, MD, within 18 months of sustaining an UE amputation. Data collection occurred from 10/18/2011 to 5/10/2014. The WRNMMC Institutional Review Board (IRB) gave approval for the study and written informed consent was obtained from all participants. Inclusion criteria consisted of the presence of an UE amputation, a normal neurological examination (except for amputation), and no prior history of vertebral disk disease/condition, sciatica, or radiculopathy. Exclusion criteria included the presence of traumatic brain injury, known uncontrolled systemic disease, significant Axis I or II diagnosis in the 6 months prior to enrollment, and a score lower than a 42/50 on the Test of Memory Malingering (TOMM).

### System components

**Figure 1:**
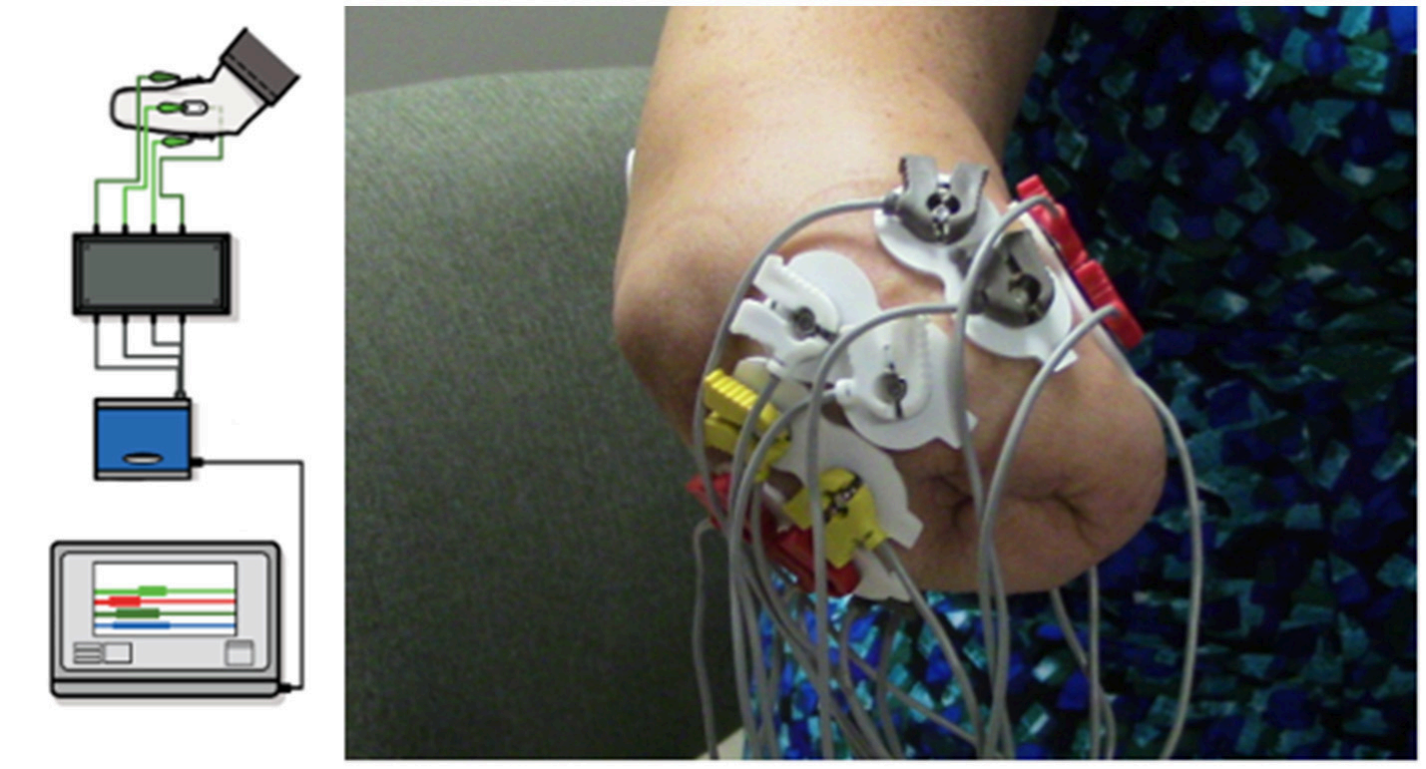
VIE system set-up and electrode configuration. Using MiniVIE open source code, created in affiliation with the John Hopkins University Applied Physics Laboratory and available at https://bitbucket.org/rarmiger/minivie, myoelectric signal processing was used to execute pattern recognition training and virtual avatar limb control. The left image illustrates the various components of the VIE platform, including live motor data collection, filtering and signal processing, pattern classification and machine learning modules, and user assessments to evaluate classifier performance (6). The right image demonstrates the circumferential placement of the eight pairs of surface electromyography electrodes around the user's residual limb with one ground electrode positioned either below the elbow or the shoulder depending on residual limb length.

The VIE system is laptop-based and contains both an operator and a visualization screen with five core sub-systems: inputs, signal analysis, controls, plant, and presentation. The input modules can process cortical inputs, surface EMG signals, and intramuscular EMG signals. Surface EMG signals were utilized as the input for this study (6, 7). A circumferential array of eight non-invasive LTI dome electrode pairs (Liberating Technology, Inc. Holliston, MA, USA) was placed around the residual limb 1 cm above the amputation site, in addition to a single ground electrode placed 1 cm below either the elbow for individuals with trans-radial and more distal amputations or 1 cm below the shoulder for individuals with trans-humeral and more proximal amputations. An electrically isolated data acquisition system was used to digitize the sEMG signals (Figure 1). Signal analysis algorithms internal to the VIE performed sEMG signal filtering, signal feature extraction, and motion classification using machine learning-based pattern recognition software. We used a Linear Discriminant Analysis (LDA) classifier. The raw sEMG data was sampled at 1,000 Hz, high-pass filtered using a 3rd order Butterworth filter with break frequency of 20 Hz, and processed into four signal features (Mean Absolute Value, Curve Length, Zero Crossings, and Slope Sign Changes). Training was done by labeling the sEMG features recorded in correspondence to each movement motion label presented graphically to the user. The system output display, based on the Musculo-Skeletal Modeling Software, rendered a stereoscopic, user motion-controlled 3-D arm, observable on the visualization screen (15). This allowed the user to control the virtual arm in real time. The VIE synchronizes with a specific physical prosthetic limb system, the Modular Prosthetic Limb (MPL). Pattern recognition-based motor classification with the VIE facilitates transition from virtual to physical limb control (16). The most recent implementation of the VIE, as used for this study, is the open-source MiniVIE code project, part of The Open Prosthetics Project (http://openprosthetics.org/). The MiniVIE code project reflects the concepts and workflow of the JHU/APL VIE platform in a separate and lightweight MATLAB-based program.

### VIE procedure

Participants were screened, enrolled, and consented by a member of the WRNMMC research team prior to study participation. Thirteen participants, representing 14 limbs, completed twenty 30-min virtual therapy sessions over the course of 1–2 months. Each session consisted of a 30-min passive period, in which the participant observed and attempted to replicate the movements of the limb of a virtual avatar (Video 1). Participants were instructed to follow along with the screen movements using their residual limb and phantom limb, if present. Surface EMG data was simultaneously recorded from their residual limbs. These signals were labeled using the cued motion of the passive virtual limb. There were 11 motions: wrist flexion, extension, pronation, and supination, as well as hand opening and several grasp patterns. At the start of each session the motions were presented in a set sequence and then switched to being presented in a randomized order. Each motion was displayed in sets of 2-min intervals. Participants were asked to additionally complete four passive sessions with their intact limb, which served as the control sessions. Two of the participants had bilateral UE amputations and one opted to complete the study two times—once with each arm. He therefore did not have a true “control” session.

The active motor control component of the VIE, or the MiniVIE system, was created by JHU/APL after the study began and offered to all participants who were enrolled after its completion. All eight of the participants who were given the option to complete the active motor control training did elect to complete it. This second training phase involved learning to drive three sets of motions within the VIE platform: Basic, Advanced, and Digit Control. These sessions lasted 30-min and immediately followed passive motor training. The individual who elected to complete the passive portion of the study with each of his amputated limbs also elected to complete this phase of the study twice. The Basic motion set included wrist rotation in/out, wrist flexion/extension, cylindrical grasp, and hand open. The Advanced motion set added lateral, spherical, and pointer grasps to the Basic set. The Digit Control set included control of each finger plus the hand open motion. Linear Discriminant Analysis (LDA) was used to identify the intended movements from the recorded data and to classify the data sets accordingly.

**Figure 2:**
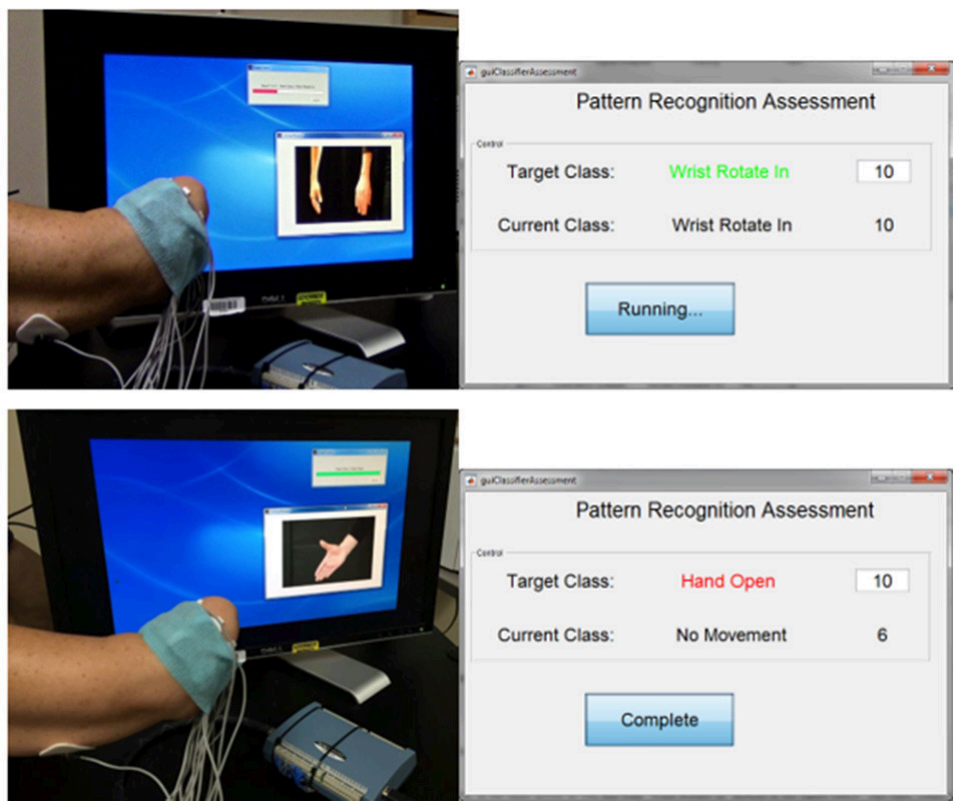
Interface for assessing controllability of the virtual limb system. The user trains a motion within the MiniVIE program by moving his phantom limb through a series of prompted motions as surface electromyography data is collected from electrodes placed on the muscles of his residual limb. The user is then assessed on his ability to reproduce these trained motion patterns by attempting to complete 10 correct readings of each target motion within a specific period of time. In the top panel, the user is training (top left) and then assessing (top right) the motion of “wrist rotate in.” The user successfully achieves all 10 motion classifications in the allotted time. In the bottom panel, the user is training (bottom left) and then assessing (bottom right) the motion of “hand open.” He completes the target motion correctly six times before the time ends.

**Figure 3:**
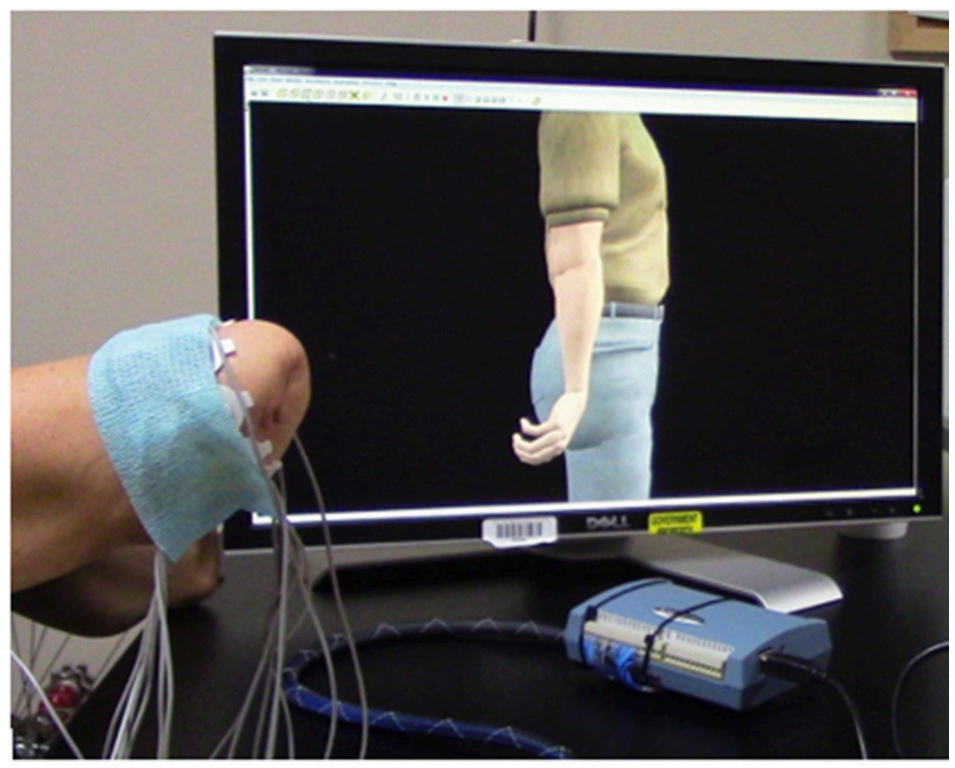
Manipulation of a virtual avatar limb within a three-dimensional framework. After training a Linear Discriminant Analysis (LDA) algorithm within the VIE training system, each user had the opportunity to direct the motion of a virtual hand and wrist during a period of free-play. Inputs were myoelectric and collected from the user's residual limb. This program provided real-time feedback on training data, as well as the opportunity to practice and improve upon pattern recognition feedback control.

Participants completed the MiniVIE training by executing movements with their phantom limb in response to computer-presented cues (Figure 2) in two sets of 2 s per motion. Unique sEMG signals associated with each intended motion were collected from the circumferential array of electrodes along the participant's residual limb. Once the LDA algorithm was trained, participants controlled the motion of a dexterous virtual hand and wrist avatar within the VIE during a period of free-play (Figure 3). This activity provided real-time feedback on training data accuracy and on each participant's ability to execute desired UE movements within the virtual environment. Seven of the eight participants who completed active VIE training also completed weekly evaluations with their intact limb that served as a “control.” The eighth participant had bilateral UE amputations and instead completed the study once with each upper limb. Training with the “control” limb was completed in an identical manner to training with the residual limb.

### VIE assessment

The analysis of passive motor training data was completed in post-processing using machine-learning algorithms. The sEMG data that had been collected continuously during the virtual sessions was identified and distinguished into discrete motion classes. These classes were labeled according to the motion of the virtual avatar at the time of data collection. These discrete, labeled motion sets were then loaded into the VIE signal classifier to generate an average motion accuracy score. We considered the trajectory of these accuracy scores across study sessions.

Unlike the passive motor training assessment, the analysis of the active motor training happened at the end of each session. These assessments quantified the user's ability to control the virtual limb using pattern recognition software and were completed within the MiniVIE system. The computer prompted the participants to perform each trained motion over a 100 ms interval (Figure 2). For each motion, they were given 5 s to achieve 10 correct motion classifications. During the assessment, the computer presented the participant with both the targeted motion class and the motion class that their data was currently being read as. This feedback allowed the participant to modify their approach in real-time as needed to achieve the target motion. These assessments generated classification accuracy scores, which reflect how well the participant in testing was able to reproduce the unique sEMG patterns they had generated in training. We defined the threshold for prosthetic acceptance as equal to or greater than a classification accuracy score of 95% based on several prior studies of myoelectric pattern recognition control of UE prostheses (12, 17–19).

### Statistical analysis

Individual analyses were conducted for passive motor scores and active motor scores (Basic, Advanced, and Digit motion sets). For each analysis, change in accuracy scores over time was assessed using unadjusted longitudinal linear mixed effects regression models. These models account for within-subject correlation of scores, inconsistent measurement intervals, and missing data (20). All tests were two-tailed and *p* = 0.05 was regarded as the threshold for significance. Analysis was accomplished using open-source statistical analysis software (R version 3.4.2).

## Results

### Participants

Fourteen individuals were recruited and consented to this study. Thirteen male, active duty military personnel between 20 and 33 years of age completed this study (Table 1). One participant withdrew after three study sessions due to scheduling conflicts. Eleven of the 13 participants had sustained unilateral UE amputations, while two had sustained bilateral UE amputations. One of the participants with bilateral UE amputations volunteered to complete the study twice, once with his right arm and once with his left arm. These two data sets were collected independently and given independent identifiers. Thus, the total sample size was 14 data sets collected from 13 individuals. For the active motor training, or MiniVIE, component of the study, nine data sets were collected from eight individuals. The same participant who opted to complete the passive training with each of his limbs also completed the active training twice.

**Table 1:** Participant demographics.

**User**	**Age (years)**	**Site, side of amputation**	**Months since amputation**	**Phantom limb pain**
01	27	ED, center	14	Yes
02	22	TH, right	9	Yes
03	27	TH, right	18	Yes
04	28	TR, center	18	Yes
05	33	TR, center	4	No
07	23	TR, center	13	Yes
08	30	WD, Right	6	Yes
09	30	WD, center	2	No
10	29	WD, center	105	No
11–L	24	TH, center	14	No
11–R	24	TR, right	14	No
12	28	PH, center	9	No
13	22	WD, right	6	Yes
14	20	TR, center	10	Yes

*The age, amputation details, and baseline phantom limb pain status for each participant are displayed in Table 1. A total of 14 participants were recruited to the study and 13 completed all sessions. Participant 06 is not reflected, as he withdrew from the study early on due to scheduling conflicts. Participant 11 has a bilateral upper extremity (UE) amputation and volunteered to complete the study twice—once with his right arm and once with his center arm. These data sets were collected and analyzed independently, and are labeled as 11_R and 11_L, respectively. The following abbreviations describe the site of UE amputation: ED, elbow disarticulation; TH, trans-humeral; TR, trans-radial; WD, wrist disarticulation; and PH, partial hand*.

### VIE results

A total of 277 passive motor training sessions were conducted. Each individual study identifier completed an average of 17.6 ± 4.7 sessions over 66.2 ± 40.5 days. The mean classification accuracy score across all sessions for all 14 data sets was 43.8 ± 10.7%. Given 11 unique motions performed during passive training, the chance of completing a motion purely by chance was 9%, which was statistically significantly different from the observed mean classification score of 43.8% (one-sample *t*-test *p*-value < 0.001). This suggests that participants were actively engaged in the virtual limb-following tasks. There was no significant change in accuracy scores for passive motor training over time (per one additional session, β-coefficient < 0.001, *p* = 0.36).

**Figure 4:**
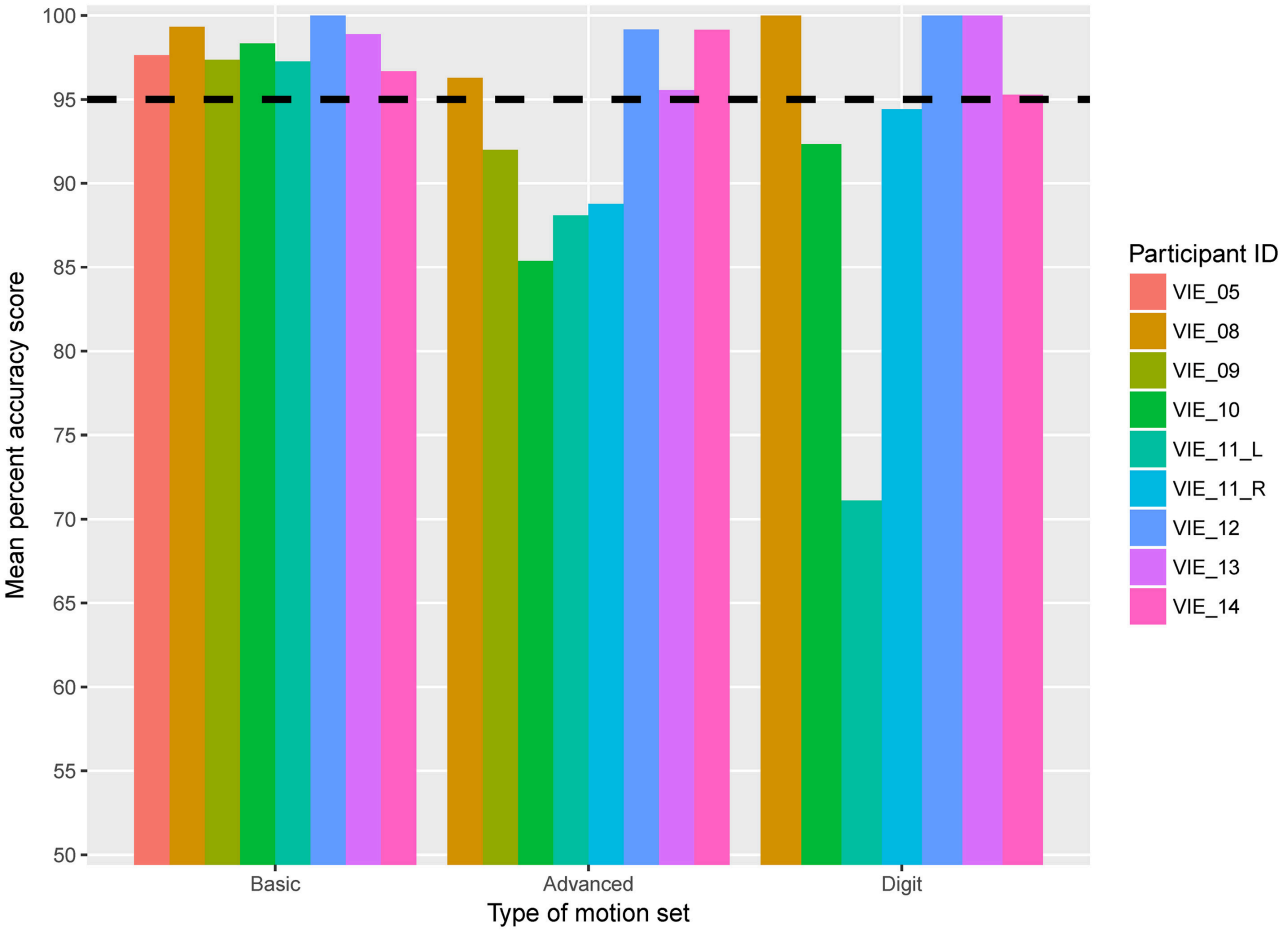
Mean active motion score by participant. Eight participants, representing nine individual data sets, completed the active motion training portion of the VIE study. The horizontal dashed line represents 95% mean classification accuracy, which we defined as the threshold for prosthetic acceptance. Each column represents an individual participant. The columns are grouped based on type of motion set: Basic, Advanced, or Digit. The y-axis begins at 50% classification accuracy to better distinguish small differences in score.

For the active motor training, or MiniVIE, portion of the study mean accuracy scores greater than 95% were achieved by all nine studied limbs for the Basic set (Figure 4, Table 2). For the Advanced set (i.e., Basic set plus three complex grasps), four of the eight participants (50%) representing five of the nine (56%) data sets achieved proficiency greater than 95%. These same four participants achieved over 95% accuracy with Digit control (Figure 4, Table 2).

**Table 2:** Active motor control, or “MiniVIE,” accuracy scores.

**User**	**P-basic**	**P-advanced**	**P-digits**	**C-basic**	**C-advanced**	**C-digits**
05	**97.6%** (16/19)	0% (0)	0% (0)	**100.0%** (6/6)	0% (0)	0% (0)
08	**99.3%** (19/20)	**96.3%** (13/20)	**100.0%** (4/4)	**100.0%** (4/4)	**100.0%** (4/4)	**100.0%** (1/1)
09	**97.4%** (23/29)	92.0% (7/14)	0% (0)	**100.0%** (8/8)	0% (0)	0% (0)
10	**98.3%** (5/6)	85.4% (1/6)	92.3% (2/5)	**100.0%** (1/1)	**100.0%** (1/1)	0% (0)
11	**97.3%** (21/28)	88.1% (2/7)	71.1% (0/3)	**96.2%** (18/20)	88.8% (6/18)	94.4% (14/17)
12	**100.0%** (20/20)	**99.2%** (18/20)	**100.0%** (20/20)	**100.0%** (4/4)	**96.1%** (3/4)	**97.1%** (3/4)
13	**98.9%** (19/21)	**95.6%** (11/17)	**100.0%** (13/13)	**100.0%** (6/6)	**98.3%** (3/4)	**100.0%** (3/3)
14	**96.7%** (20/25)	**99.1%** (12/13)	**95.3%** (4/6)	**100.0%** (5/5)	**97.0%** (2/3)	**100.0%** (1/1)

*The motion classification accuracy results for the active motor control training, or “MiniVIE,” portion of the VIE study are displayed in Table 2. Included are each participant's mean accuracy scores for the Basic, Advanced, and Digit motion sets achieved while using either their residual/phantom (P) limbs or intact/control (C) limbs. The scores that pass the threshold for prosthetic efficiency (i.e., 95% accuracy) are bolded. The fractions provided within parentheses represent the number of assessments where that participant achieved greater than 95% accuracy over the total number of assessments they completed under those testing conditions. Of note, participant 11 has bilateral upper extremity amputations and opted to complete the study twice—once with each arm. Therefore, for participant 11 both the left and the right columns are “P” data, as he does not have a true intact, or “control,” limb. The left and right columns correspond to the data collected from his right and left arms, respectively*.

For both the Basic and Advanced sets, there was a significant improvement in motor accuracy scores over time (per additional session: β-coefficient 0.125, *p* = 0.022; β-coefficient 0.45, *p* = 0.001, respectively), and there was a trend toward significance for the Digit set (β-coefficient 0.594, *p* = 0.077). Improvement in motor accuracy scores occurred early; the mean accuracy score for the nine arms completing the Basic set increased from 90.2% (range: 76.7–100%) to 100% (range: 95.8–100%) across the first three sessions.

All participants who completed the weekly “control” arm sessions within the MiniVIE platform (i.e., 7 of the 8 participants, representing 7 of the 9 independent data sets) attained 100% accuracy with their intact limbs for the Basic set. It is notable that even with their intact limbs, these participants were unable to achieve perfect results with either the Advanced or Digit sets (Table 2). Interestingly, the eighth participant who had sustained bilateral UE amputations demonstrated similar accuracy scores regardless of which arm was being tested. With either limb, he achieved greater than a 95% mean accuracy score with the Basic set, less than a 95% mean accuracy for the Digit set, and mean accuracy scores that fell within one percentage point for the Advanced set (88.1 and 88.8%) (Table 2).

## Discussion

In the passive portion of this study, 13 participants (representing 14 independent data sets) demonstrated the ability to move their residual and phantom limbs in concert with a virtual avatar and elicit sEMG signals unique to those motions. The variability in results in this portion of the study is likely due to the randomized nature of the motion generator, which made it difficult for participants to precisely control the onset and offset of their movements. The low accuracy scores are further explained by the lack of user feedback during sessions, which restricted the participants' ability to edit their contraction patterns in real-time. The main conclusion to be drawn from the passive control results is that the participants were engaged in the task of following the virtual limb, as indicated by all of the accuracy scores being better than chance (43.8% observed; 9% expected purely by chance; one-sample *t*-test *p* < 0.001).

When given the opportunity to train a pattern recognition-based myoelectric arm controller, eight individuals (representing 9 independent data sets) demonstrated proficiency with wrist motions, hand grasps, and individual finger control. All participants who completed the active motor portion of this study achieved classification accuracy scores for six individual movements that passed the threshold for prosthetic use, which we defined as 95% or greater. Four of these individuals (representing five independent data sets) achieved greater than 95% accuracy for a total of 14 movements (i.e., the Basic, Advanced, and Digit Control motion sets). Furthermore, results from longitudinal mixed effects regression models demonstrate that there is a cumulative learning effect from repeated training: participant scores improved with each subsequent training session. These results demonstrate that individuals with UE loss can effectively learn to create and reproduce unique sEMG contraction patterns for a variety of UE motions.

Though there is a paucity of literature quantifying and characterizing changes in motor accuracy following limb loss, Sebelius et al. observed that six individuals with unilateral trans-radial limb loss actuated seven discrete motions with a virtual computerized hand (21). The hand was operated through sEMG-mediated, thought-based control of an Artificial Neural Network (ANN), as well as a data glove worn on the contralateral healthy hand. There were no significant differences in control with respect to time since amputation over seven sEMG training sessions. A study by Schabowsky et al. examined the motor accuracies of eight trans-radial prosthetic users and eight neurologically intact persons without amputation in executing reaching movements within the horizontal plane (22). This study used a robotic manipulandum and exposure to curl field perturbances imposed by robotic motors to determine the rate and quality of adaptation between the two groups. Adaptation was reflected by a decrease in peak error between observed and ideal trajectories. It was observed that during the late phase of adaptation (i.e., trials 36–120), error magnitude and variability were greater in the prosthetic user group compared to controls, but that overall, motor adaptation to curl fields and accuracy during reaching is similar between individuals with UE amputation and healthy individuals. These studies suggest that individuals with UE amputation have the neurorehabilitative potential to learn to manipulate virtual movements using sEMG control and to adapt to motion stimuli in virtual environments. In addition, the rapidity (total number of training sessions required) with which participants reached near perfect accuracy in movement control suggests that the brains of persons with amputation retain the ability to rapidly adapt over time. The results of our study agree with these findings, by demonstrating that motor accuracy scores for individuals with UE loss training within a virtual environment can pass the threshold for prosthetic usage (i.e., 95% accuracy) for every user and pass it rapidly (here, within the first three of 20 sessions).

The limitations of our study include the small sample size and short testing time with each participant, which restricted both the number of data points available for analysis and the learning period in which participants could master the motor control system. The limited training period made it difficult to fully characterize the timeframe and pattern of motor learning following amputation, as participants were observed during only 1–2 months of their recovery. Lastly, while virtual training systems allow us to assess a participant's ability to learn and utilize pattern recognition software for sEMG-driven control of a virtual limb, distinct differences exist between operating a virtual avatar and manipulating a physical prosthesis. Notably, our participants were not faced with the postural kinetic and kinematic challenges that come with physical prosthetic training and use. Therefore, our ability to predict how success within the VIE platform translates to control of an advanced myoelectric device is limited.

The modular design of the VIE system is a major strength, as it allows for the frequent manipulation of each subsystem and graphic limb simulation (23). While other modular neuroprosthetic frameworks exist, they are not designed specifically for operating neurally-controlled prosthetic limbs (24). Alternative virtual systems for myoelectric training may also include realistic virtual avatars and practice with real-time virtual environments of virtual tasks such as clothespin movement, posture matching, and Fitts-law target acquisition, as well as the abstraction of myoelectric signals in gaming (19, 25–27). In addition to the successful use of the VIE platform by participants with unilateral UE amputation, the ability of two participants with bilateral UE amputation to demonstrate passive motor control—and active motor control by one of these individuals—suggests the viability of the VIE system as a motor training platform for persons with bilateral UE amputation. Though many studies have cited the benefits of prosthetic training within a virtual environment, there is a dearth of literature on the comparative strengths and weaknesses of existing virtual prosthetic training systems. Additional research is needed to investigate the relative merits and limitations of these platforms, as well as the VIE, with respect to their ability to accurately characterize participant motor learning and predict future success with controlling a physical myoelectric prosthesis.

Overall, these motor results demonstrate the potential for the VIE to be used as a motor classification and training tool for operation of advanced myoelectric prostheses, such as the MPL (7). The motor training afforded by the VIE system is consistent with previous findings regarding the potential to improve and facilitate motor learning after amputation using virtual prosthetic training systems over time. Though the VIE system was designed to synchronize specifically with the MPL, successful training within the VIE system—with respect to accurate motion classification and pattern recognition-based discrimination between movement classes—may facilitate transition to and use of other advanced myoelectric prostheses that similarly utilize pattern recognition software. The MPL is an experimental device not currently available for commercial use and dissemination. A study is now ongoing to determine the functionality of the MPL and includes a VIE-based training and classification accuracy screening stage that precedes user control of the physical prosthesis. Future research can be conducted to investigate exactly how predictive success within the VIE system is for success controlling an advanced, dexterous UE prosthesis like the MPL.

The VIE platform is an effective training platform for individuals with UE amputation to learn pattern recognition-based motor control. Understanding this control paradigm may lead to improved operation of advanced myoelectric prostheses, such as the MPL. If we are to increase the current low rates of prosthetic acceptance, then we need to continue to develop training platforms that allow for the intuitive motor control of dexterous prostheses. Future research is needed to explore to what degree success within a virtual training system, such as the VIE, translates into improved functioning and quality of life for individuals with UE loss.

## Ethics statement

This study was carried out in accordance with the recommendations of the Walter Reed National Military Medical Center (WRNMMC) Institutional Review Board (IRB) with written informed consent from all subjects. All subjects gave written informed consent in accordance with the Declaration of Helsinki. The protocol was approved by the WRNMMC IRB.

## Author contributions

BP led protocol development, study administration, data analysis, and manuscript writing. RA led technical support and study design and contributed to data analysis. KY contributed to manuscript writing. AA contributed to data analysis and figure production. CM assisted with technical support. MW and KM assisted with protocol development and study administration. PP oversaw study design and study administration. JT oversaw data analysis and manuscript writing.

### Conflict of interest statement

The authors declare that the research was conducted in the absence of any commercial or financial relationships that could be construed as a potential conflict of interest.
